# A Novel Sero-Specific-Gene Dependent Multiplex PCR Enhances the Discrimination of Major *Listeria monocytogenes* Serovars

**DOI:** 10.4014/jmb.2411.11081

**Published:** 2025-03-07

**Authors:** Jing Wang, Jingliang Qin, Bin Hu, Zixu Zhang, Boyang Cao, Xi Guo

**Affiliations:** 1 The Key Laboratory of Molecular Microbiology and Technology, Ministry of Education, 23 Hongda Street, TEDA, Tianjin, P.R. China; 2 TEDA Institute of Biological Sciences and Biotechnology, Nankai University, 23 Hongda Street, TEDA, Tianjin, P.R. China; 3 Shandong Center for Disease Control and Prevention, 16992 City Ten Road, Jinan 250014, Shandong, P.R. China

**Keywords:** Foodborne pathogen, *Listeria monocytogenes*, serovar, sero-specific gene, multiplex PCR

## Abstract

*Listeria monocytogenes* is a foodborne bacterial pathogen distributed worldwide. Serotyping is extensively applied in the classification of *L. monocytogenes* and is crucial in the early stage of epidemiological tracing. Among the 13 serovars, 1/2a, 1/2b, 1/2c, and 4b are the ones most frequently isolated. Numerous PCR-based methods have been presented, however, their target genes, *lmo0737*, *ORF2110* and *ORF2819*, are prone to horizontal transfer or loss in certain strains, thus leading to incorrect serovar designation. Herein, we selected novel sero-specific genes and developed an improved multiplex PCR assay. The specificity of our assay was confirmed by the use of target and nontarget *Listeria* reference strains, as well as by the use of isolates yielding incorrect profiles in previous studies. Sensitivity tests indicated that a minimum of 5 ng of genomic DNA or approximately 3 × 10^6^ CFU of pure culture could be detected. Many collected isolates and genomes of global isolates were used to evaluate the specificity and reproducibility of our assay. The agreement between our assay and the agglutination test was 95%, and the one between our assay and the Doumith scheme was 97%. However, our assay overcomes the drawbacks of currently used PCR-based approaches by exhibiting 100% accuracy for certain strains and clones, for instance, ST782 within the hypervirulent CC2 and ST218 that were incorrectly assigned by the Doumith scheme. In conclusion, the developed assay herein will be a powerful tool and an alternative for the classification of *L. monocytogenes* strains in foodborne outbreak investigations and surveillance programs.

## Introduction

*Listeria monocytogenes* is a Gram-positive, facultative intracellular foodborne bacterial pathogen which is the causative agent of listeriosis [1]. Individuals who are immunocompromised, elderly individuals, pregnant women, and fetuses are at high risk for listeriosis [2]. The outcomes of listeriosis can be severe, and this disease has a high mortality rate (20 to 30%), making it an important public health concern [3]. Contaminated foods, including raw staple foods and ready-to-eat processed foods, are the primary source of infection for both sporadic and epidemic listeriosis cases [4].

Due to the significance of *L. monocytogenes* characterization for epidemiological investigations, numerous subtyping methods have been reported for this important foodborne pathogen [5-9]. During the last years, WGS-based approaches have provided insights for the characterization of *L. monocytogenes* with much higher resolution and have helped elucidate epidemic events [7, 10, 11]. However, as the choice of reference genome can significantly influence subsequent analyses, the single-nucleotide polymorphism (SNP)-based WGS analysis is difficult to be standardized and may be difficult to be interpreted [7, 12]. The core genome MLST method relies on well-defined standard sets of hundreds of genes, however, there are financial and technical barriers for its application, such as the high cost of sequencing and the need for bioinformatics expertise and expensive server [13]. In particular, the lengthy turnaround time of high-quality DNA extraction and library preparation limit the application of WGS in the early stages of epidemiological investigations.

Based on serological reactions against specific somatic antigens (O-antigens; it should be noted that wall teichoic acid (WTA) represents the O-antigen of *Listeria*) and flagellar antigens (H-antigens), *L. monocytogenes* has been divided into 13 serovars [14]. Recently, another newly designated serovar, 4h, which causes severe ovine listeriosis outbreaks and is highly virulent, was reported [15]. Among these serovars, at least 95% of the isolates that originated from foods and patients were of the 1/2a, 1/2b, 1/2c and 4b serovars [16]. On the other hand, phylogenomic analysis can classify *L. monocytogenes* into four divergent lineages, and a novel hybrid sublineage comprising only 4h strains. A majority of *L. monocytogenes* strains are allocated to lineage I and lineage II, with the former including serovars 1/2b, 3b, 4b, 4d, 4e and 7 and the latter consisting of 1/2a, 1/2c, 3a and 3c [15].

Serotyping is widely used to characterize *L. monocytogenes*, as this is crucial in the early stage of epidemiological tracing. The traditional serotyping method that relies on agglutination reactions is accurate but time and labor intensive [17]. Serotype-specific antibody-based enzyme-linked immunosorbent assay (ELISA) approaches have also been employed [18]. To overcome the drawbacks of the agglutination-based traditional serotyping method, a great number of molecular approaches have been established, overall showing advantages over the agglutination test [19-22]. Among them, the multiplex PCR assay that could characterize *L. monocytogenes* serovars 1/2a, 1/2b, 1/2c and 4b was developed by Doumith and colleagues (the Doumith scheme, DS) in 2004 and has since been a standard tool for molecular serotyping of *L. monocytogenes* [20]. All these assays utilize the lineage II-specific gene *lmo0737* to define serovars 1/2a and 1/2c and lineage I-specific gene *ORF2110* to define serovar 4b. In addition, *ORF2819* was designed to distinguish serovar 1/2b from other *L. monocytogenes* strains in the DS. However, a recent study noted that due to horizontal gene transfer and loss of sero-specific genes (*lmo0737*, *ORF2110* and *ORF2819*), the use of the DS can lead to incorrect serovar designations, especially when characterizing strains of some given sequence types (STs), including ST782 within the hypervirulent clonal complex 2 (CC2) [23]. This indicates a drawback associated with the design of target genes in relevant methods, and the development of novel assays with increased accuracy is needed.

Herein, to overcome the limitations of the current methods, we aimed to establish an improved multiplex PCR assay targeting novel sero-specific genes that could be used to characterize the major *L. monocytogenes* serovars with increased reliability and reproducibility. Using this method, *L. monocytogenes* 1/2a, 1/2b, 1/2c and 4c can be distinguished specifically from isolates of various genotypes.

## Materials and Methods

### Bacterial Strains

All strains used in this study were listed in Table 1. Among them, 23 *L. monocytogenes* reference strains covering 12 serovars were used to establish the multiplex PCR system. Moreover, five other *Listeria* strains, including *L. ivanovii*, *L. grayi*, *L. seeligeri*, *L. welshimeri*, and *L. innocua*, were used for specificity assessment as negative controls. Additional isolates were collected to evaluate the practicality of our assay. All strains were grown in brain heart infusion broth overnight at 37°C under constant shaking.

### Serotyping

Eight-one collected *L. monocytogenes* isolates were serotyped with a commercially available serotyping kit purchased from Denka Seiken Co. (Japan) according to the manufacturer’s protocol.

### Genomic DNA Extraction

To obtain pure genomic DNA, 1.5 ml of overnight bacterial culture was collected and then treated with a DNA extraction kit (Cat. No 4992448, Tiangen, China) according to the manufacturer's instructions.

To obtain the crude DNA extract, 1.5 ml of overnight bacterial culture was collected and washed twice with phosphate-buffered saline. Then, the pellet was mixed thoroughly with sterile distilled water containing 20 mM Tris-HCl (pH 8.0), 2 mM EDTA, and 1.2% Triton X-100 and incubated at 37°C for 10 min. Finally, the suspension was heated at 99°C for 10 min and centrifuged at 7,200 ×*g* for 1 min. The resulting supernatant was used as the DNA template for the next experiment.

### Target Gene Selection and Primer Design

The *hlyA* gene, which is specific to the species *L. monocytogenes*, was targeted as an internal control [24]. The *gttA* gene, which encodes a WTA glycosyltransferase responsible for galactose modification on WTA of *L. monocytogenes* serovar 4b [25], was selected as the candidate gene for targeting 4b. The gene *lmo0701*, located in the flagellar gene cluster of serovar 1/2a, and its homolog *A409_0759*, mapped in the flagellar gene cluster of serovar 1/2b, were selected as candidates targeting serovars 1/2a and 1/2b, respectively. The *lmo1118* gene, which was used to differentiate 1/2c strains from other serovars in a previous study, was used to characterize the same serovar in our assay. All primers were designed based on the above sero-specific candidate genes using Primer Premier v5.0 software. The specificity of all the designed primers was preliminarily examined by comparing their sequences against the GenBank database using BLAST.

### Development of a Multiplex PCR Assay

A multiplex PCR assay with only a single reaction was designed and used to detect *L. monocytogenes* serovars 1/2a, 1/2b, 1/2c, and 4b. Each PCR was carried out in a 20 μl mixture containing 1×Multiplex PCR StarMix (ZA092-101, Genstar, Suzhou, China), 200 to 400 nM each of the respective primers (Table 2), and 50 ng of pure genomic DNA as template. PCR amplification was performed on a thermocycler using the following parameters: initial denaturation at 95°C for 3 min; 30 cycles of denaturation at 95°C for 30 s, annealing at 60°C for 3 min, and extension at 72°C for 90 s; and a final extension at 72°C for 10 min. Two microliters of each PCR product was subjected to 1.5% agarose gel electrophoresis and visualized by GelRed (Biotium Inc., USA) staining. Then, images were acquired using a gel imaging system (Tanon 4200, China).

### Sensitivity of the multiplex PCR Assay

To evaluate the limit of detection for purified bacterial DNA, serial dilutions of genomic DNA (50 ng to 5 fg) from each *L. monocytogenes* serovar were prepared. Multiplex PCR was performed using each 10-fold serial dilution as a template.

To evaluate the limit of detection of the assay for pure cultures, the culture of each serovar of *L. monocytogenes* was subjected to 10-fold serial dilution to concentrations ranging from approximately 10^9^ to 10^2^ CFU/ml, and counts were estimated by the plate-counting method. Then, 3 μl of DNA crude extract from each diluted culture was used as a template for subsequent PCRs.

## Results

### Analysis of Sero-Specific Candidate Genes

Given that the antigenic difference among *L. monocytogenes* serovars is determined by both WTAs and H-antigens, we screened the genes associated with WTA and flagellum synthesis to investigate whether any candidate sero-specific gene could be selected. Structurally, the WTAs in *L. monocytogenes* can be divided into 2 types: strains of serovar 1/2, 3, and 7 possess the type I WTA, and those of serovar 4 belong to type II WTA [26]. Accordingly, the WTA gene clusters within each type are conserved, but can be distinguished between serovars representing different WTA types [15]. *gttA*, located in the type II WTA gene cluster, encodes a glycosyltransferase responsible for the galactose modification on WTA of *L. monocytogenes* serovar 4b [25], and was selected as a candidate serovar 4b-specific gene. Since the WTA structures and WTA gene clusters of serovars 1/2a and 1/2b are identical, it is proposed the antigenic difference of them was relevant to their distinct flagellar structures. Thus, we further aligned the sequences of the flagellar gene clusters of both serovars 1/2a and 1/2b. Although they showed very high overall identity, a pair of homologous genes (*lmo0701* in 1/2a and *A409_0759* in 1/2b) that shared relatively low nucleotide identity (87%) were discovered, suggesting that these genes could serve as sero-specific candidates for serovars 1/2a and 1/2b, respectively (Fig. S1). To characterize serovar 1/2c, we retained the *lmo1118* gene as the target, which has been applied to differentiate 1/2c from other serovars in previous studies [20, 22].

**Fig. 1:**
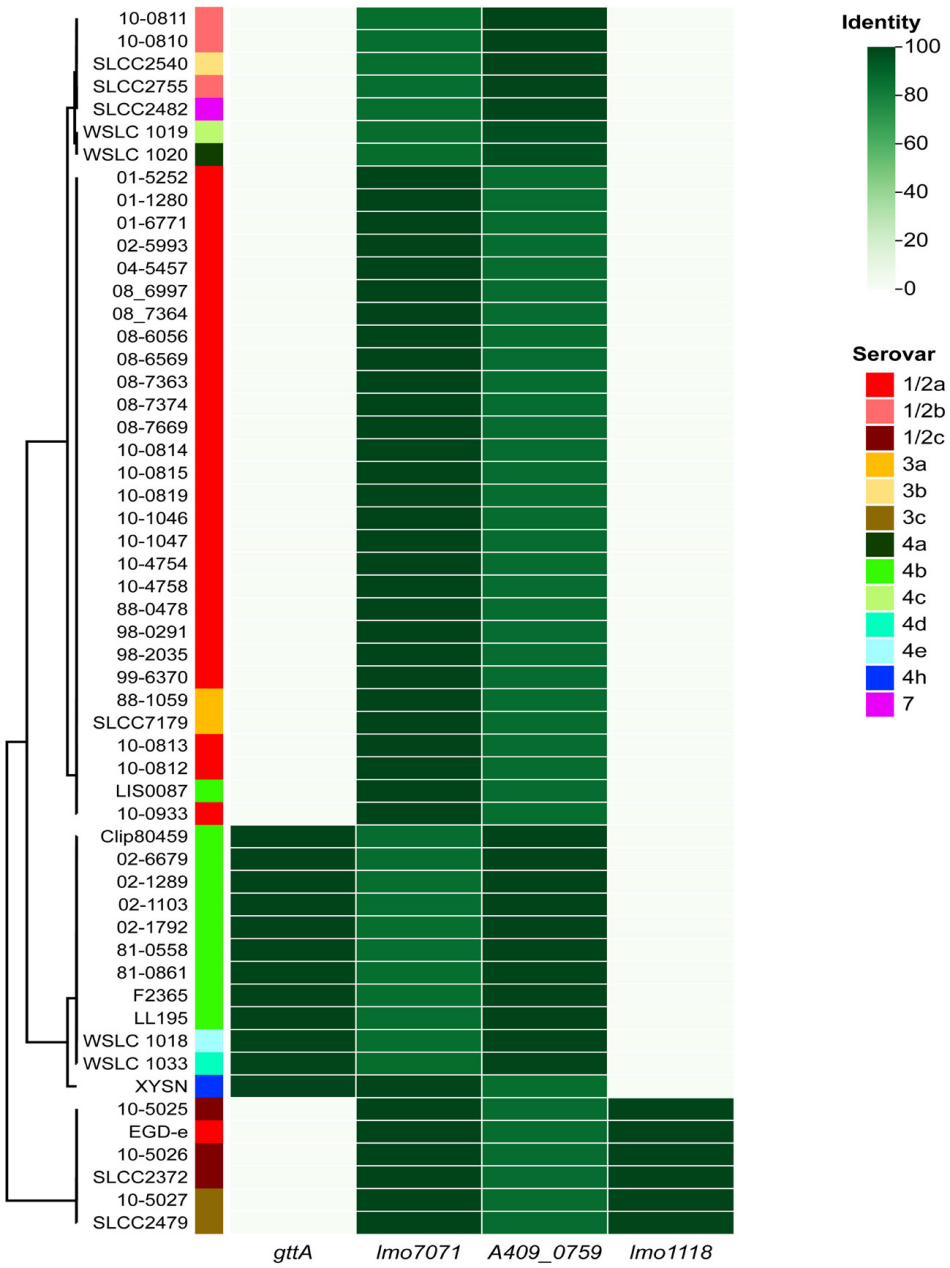
Heatmap showing the distribution of sero-specific candidate genes among isolates covering 13 serovars whose genomes have been completely sequenced and assembled. The isolates were clustered according to the gene allocation, with their serovars depicted by different color blocks.

Next, the above candidate sero-specific genes were compared against closed genome sequences downloaded from the GenBank database covering 13 serovars (Fig. 1). The candidate serovar 4b-specific gene *gttA* was distributed in almost all the 4b strains examined except one (LIS008) and was shared with 4d, 4e, and 4h. Furthermore, *lmo0701* and *A409_0759* could clearly differentiate strains assigned to 1/2a and 1/2b, although each gene was present in several other minor serovars. In addition, the analysis also indicated that *lmo1118* is specific to serovar 1/2c, as *lmo1118* was found only in strains assigned to serovar 1/2c and the minor serovar 3c, as well as a serovar 1/2a strain, EGD-e, as an exception. The atypical feature of EGD-e has been revealed by a previous marcoarray hybridization study, which indicated that the global genome structure of EGD-e is more similar to that of strains of the serovar group 1/2c-3c than to strains of 1/2a-3a [27]. Therefore, in summary, these genes, singly or in combination, provided enough power to discriminate the major *L. monocytogenes* serovars and exhibited potential applicability in the development of molecular-based assays.

### Specificity of the Multiplex PCR Assay

**Fig. 2:**
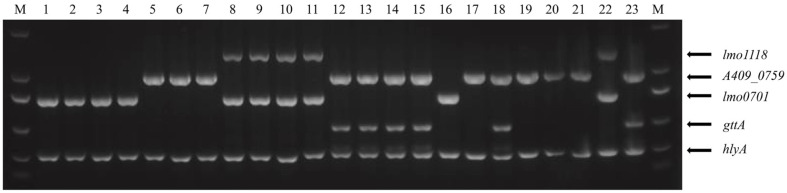
Agarose gel electrophoresis of PCR products for the *L. monocytogenes* reference strains listed in Table 1. M, DL2000 DNA standard; lane 1, CMCC 54001 (1/2a); lane 2, NCTC 7973 (1/2a); lane 3, NCTC 7974 (1/2a); lane 4, ATCC 19111 (1/2a); lane 5, NCTC 10887 (1/2b); lane 6, ATCC BAA-3153 (1/2b); lane 7, ATCC BAA-3135 (1/2b); lane 8, NCTC12427 (1/2a); lane 9, ATCC 19112 (1/2c); lane 10, NCTC 9862 (1/2c); lane 11, ATCC 51779 (1/2c); lane 12, CMCC 54007 (4b); lane 13, NCTC 9863 (4b); lane 14, NCTC10527 (4b); lane 15, ATCC 13932 (4b); lane 16, ATCC 19113 (3a); lane 17, NCTC 4883 (4c); lane 18, ATCC 19117 (4d); lane 19, NCTC 10890 (7); lane 20, CMCC 54006 (4a); lane 21, CIP 78.35 (3b); lane 22, CIP 78.36 (3c); lane 23, ATCC 19228 (4e).

The multiplex PCR assay was tested using reference *L. monocytogenes* strains covering 12 serovars and five reference strains representing other *Listeria* species (Table 1). As shown in Fig. 2, all *L. monocytogenes* strains generated amplicons corresponding to *hlyA*, which is specific to this species, and simultaneously, each strain generated the amplicon(s) of the expected size relative to the targeted sero-specific genes, except the serovar 1/2a strain EGD-e, NCTC12427, which generated the 1/2c profile. Moreover, none of the strains belonging to other *Listeria* species generated any PCR products (data not shown). This result confirmed the reliability of our assay.

As reported by Brown *et al*., *L. monocytogenes* strains of a few given sequence types (STs) or clonal complexes (CCs) cannot be serotyped correctly using the DS due to gene transfer and loss events [23]. To further test the specificity of our assay and compare it to that of the DS, we downloaded the genomes of isolates studied by Brown *et al*. and performed *in silico* PCR. The results generated from 39 strains representing 10 STs/9 CCs showed that, in contrast to the DS which lead to the incorrect or confusing designation, serovar assignment of our assay was completely consistent with those obtained from whole-genome sequencing (WGS) and/or agglutination tests (Table 3), thus indicating that the present study enhanced the discrimination of major *L. monocytogenes* serovars by targeting the novel sero-specific-genes.

### Sensitivity of the Multiplex PCR Assay

Serial 10-fold dilutions of purified bacterial genomic DNA from *L. monocytogenes* serovars 1/2a, 1/2b, 1/2c, and 4b with amounts ranging from 50 ng to 5 fg were used as templates to carry out the PCR. For serovars 1/2a, 1/2b and 1/2c, 0.5 ng of genomic DNA was sufficient to generate the expected amplicons, while for serovars 4b, the limit of detection was 5 ng of genomic DNA (Fig. S2).

The sensitivity was also assessed using pure cultures of *L. monocytogenes* serovars 1/2a, 1/2b, 1/2c, and 4b. PCR results indicated that each serovar could be detected at a level of 3 × 10^6^ CFU (Fig. S3).

### Testing of the Assay in Our Collected Isolates

Multiplex PCR and agglutination assays were used to screen the serovars of collected isolates in this study. According to the developed assay, 20 isolates were assigned to serovar 1/2a, 12 isolates to 1/2b, 46 isolates to 1/2c, and three isolates to 4b. Meanwhile, the serovar allocation by the agglutination tests was as follows: 21 isolates were serovar 1/2a, 11 were 1/2b, 45 were 1/2c, three were 4b, and one was untypeable. Taken together, the agreement of the results for these isolates between the two assays was 95.1%. The high consistency rate indicates the potential of the improved multiplex PCR in supplementing or even replacing the traditional agglutination test in routine diagnostic and epidemiological applications, which is labor and time consuming.

### Testing of the Assay with Genomes of Global Isolates

To further evaluate the specificity and reproducibility of our assay, 32 *L. monocytogenes* genome assemblies and sequencing reads covering 127 *L. monocytogenes* isolates were downloaded from the BIGSDB database (https://bigsdb.pasteur.fr/). These isolates, whose serovars via agglutination tests were available from the submitters, covered 60 STs and 40 CCs, reflecting a board range of sources (Table S1). With the sequencing reads, they were assembled using our in-house program. Then, the genomes of the 159 isolates were subjected to *in silico* multiplex PCR. Our test indicated that out of the 159 isolates, the results for 142 isolates generated by our assay were consistent with those generated by agglutination tests, showing 89% consistency, and consistency between our assay and DS was 97% (155 out of 159).

## Discussion

Molecular-based serotyping assays have been applied in the classification of a great number of bacterial pathogens. Normally, the sero-specific target genes used are located in and selected from the O-antigen gene clusters, the capsular gene clusters and/or the flagellar gene clusters, whose coding enzymes are responsible for the synthesis of O-antigens, capsules, and/or flagella, all of which are antigenic determinants [28-31]. Among the current *L. monocytogenes* serotyping methods, the targets are phylogenetic lineage-specific genes, mainly including *lmo0737* and *ORF2110*. The occurrence of horizontal transfer and loss of *lmo0737* and *ORF2110*, as well as of *ORF2819*, can result in incorrect serovar characterization with DS [23]. It is reasonable to deduce that this will be the case when using other current molecular-based assays. In addition, it is proposed that the use of lineage-specific genes as sero-specific targets may be attributed to the very high nucleotide similarities among the WTA and flagellar gene clusters of different *L. monocytogenes* serovars. However, through further analysis, novel sero-specific candidates within two clusters (*gttA* located in the WTA gene cluster of serovar 4b and *lmo0701* and *A409_0759* located in the flagellar gene clusters of serovars 1/2a and 1/2b, respectively) were screened and selected by us, and their potential in molecular serotyping via *in silico* analysis were demonstrated. Since these genes are directly involved in the synthesis either WTA or flagellum, the antigenic determinants of *L. monocytogenes*, variation in these genes can lead to the change of antigenic profile, and keep the correspondence between genotype and phenotype. In addition, no mobile elements surrounding the WTA and flagellar gene clusters and relevant gene transfer events have been reported, indicating *gttA*, *lmo0701* and *A409_0759* can be used as reliable serotyping markers.

As shown in our results, the serotyping agreements between our assay and agglutination tests and between our assay and DS were both great, with the latter being a little higher than the former (89% vs 97%), when investigating the global isolates (Table S1). However, there were still a few isolates generating the contradictory results among these methods, which were discussed in detail as below: 1), according to further analysis, the inability of our assay and DS to serotype six of the isolates resulted from the lack of the *hylA* gene, the species marker for *L. monocytogenes* used in the present study. The fast K-mer algorithm (KmerFinder) [32] and our analysis based on genomic data showed that , three of them were *L. ivanovii*, and the other three were *L. innocua*, rather than *L. monocytogenes* (Table S2). This conclusion was correlated well to the MLST analyses against the six isolates. The analysis on these non-*L. monocytogenes* strains demonstrated convincingly the use of *hlyA* gene as an internal control, which can enhances the overall accuracy and reliability of the multiplex-PCR system.; 2), for additional ten isolates whose serovar assignments were consistent only between our assay and the DS, we hypothesize that the serovars of these isolates were assigned incorrectly by the agglutination tests; and 3), it is especially noticed that all four ST218 strains from Brazil and Italy were assigned to serogroup IVb-v1 via DS, but were designated as serovar 4b by our assay (one of them was shown 1/2c via agglutination test, which we propose may be serotyped incorrectly.). This is because that these atypical 4b strains harbor the lineage II-specific *lmo0734*-*lmo0739* cassette (*lmo0737* is located in this region) via horizontal gene transfer, thus giving the IVb-v1 profile using DS, which has been evidenced in previous studies [33, 34]. And the occurrence of other ST218 strains showing IVb-v1 serogroup has also been reported in Brazil [35]. In general, the isolates used for comparison were widely representative, and the ability of totally correct serovar differentiation demonstrated the robustness of our assay in characterizing isolates of diverse genetic background.

Apart from the overall good consistency with DS and agglutination test for the characterization of most isolates, the improved multiplex PCR assay also exhibited better discriminating power for certain strains and clones, for instance, ST782 within the hypervirulent CC2 and some newly emerging STs, than currently used PCR-based approaches (Table S2). The strains whose DS serovar assignments were in conflict with those generated from agglutination tests and our assay were isolated from clinical, environmental and animal specimens, but only from USA. Although a few ST218 strains showing DS IVb-v1 were isolated from other countries, such as Brazil, isolates from more locations need to be collected to further verify the present assay, especially those with abnormal DS profiles.

The assay's inability to distinguish rare serovars, such as 3a, 3b, 3c, 4a, 4c, 4e, 4d, and 7, is a limitation of the present study. However, compared to 1/2a, 1/2b, 1/2c and 4b, which are of prevalence in samples from both human listeriosis and foodborne outbreaks, these uncommon serovars were very rarely isolated. Therefore, we still focus on the detection of the four major serovars, which is the most suitable for the surveillance and epidemiological purpose. WTA, which is one of the main antigenic determinants of *L. monocytogenes*, is structurally classified into two groups, and the WTA structures of different serovars within each group are very similar, with only slight differences that are mainly attributed to the displayed sugar substituents [36]. For example, 4b strains can be distinguished from other type II WTA serovars by its glucose and galactose decoration. Thus, it is proposed that revealing the genetic basis for WTA decoration may provide more candidates for sero-specific genes, and indeed, *gttA*, which is required for WTA glycosylation in serovar 4b [25], was proved to be effective in the present study. Therefore, more WTA synthesis associated genes should be investigated in detail, including those within rare serovars, which may exhibit the potential as sero-specific candidates, and expand the assay’s applicability and utility in serotyping.

## Supplemental Materials

Supplementary data for this paper are available on-line only at http://jmb.or.kr.



## Figures and Tables

**Table 1:** Listeria strains used in this study.

Strains^a^	Serovar	Total number
*L. monocytogenes* reference strains		23
CMCC 54001, NCTC 7973, NCTC 7974, ATCC 19111, NCTC12427 (EGE-e)	1/2a	5
CMCC 54007, NCTC 9863, NCTC10527, ATCC 13932	4b	4
CMCC 54006	4a	1
NCTC 10887, ATCC BAA-3153, ATCC BAA-3135	1/2b	3
ATCC 19112, NCTC 9862, ATCC 51779	1/2c	3
ATCC 19113	3a	1
NCTC 4883	4c	1
ATCC 19117	4d	1
NCTC 10890	7	1
ATCC 19228	4e	1
CIP 78.35	3b	1
CIP 78.36	3c	1
Other *Listeria* strains		5
*L. ivanovii* ATCC BAA-678		1
*L. grayi* ATCC 19120		1
*L. seeligeri* ATCC 35967		1
*L. welshimeri* ATCC 35897		1
*L. innocua* ATCC 33090		1
Our collected *L. monocytogenes* strains		81

^a^CMCC, National Center for Medical Culture Collections, China; ATCC, American Type Culture Collection; NCTC, National Collection of Type Cultures, United Kingdom; CIP, Biological Resource Center of Institut Pasteur.

**Table 2:** Characteristics of primers sets used in this study.

Target gene	Primer sequence (5’-3’)	Product size (bp)	Serovar specificity	Accession no.	Primer concentration (nM)
*hlyA*	CAGGAGTTCCCATTGCTTA	261	*L. monocytogenes*	NC_003210	200
	TGGACGATGTGAAATGAGC				200
*gttA*	CATATTCAGATTGTGTCGTGTC	502	4b, 4d, 4e, and 4h	NZ_CP007210	400
	TTTTGAACAATGGCATAAAT				400
*lmo0701*	CCTGGAACCTCCGATGAT	761	1/2a, 1/2c, 3a, and 3c	NC_003210	200
	TTGCTGTTTTTTGCCATAA				200
*A409_0759*	ATCCTGAACCCCCGATAA	1008	1/2b, 3b, 4a, 4b, 4c, 4d, 4e, and 7	CP007168	200
	CTGCACTTGTGAAACGGTT				200
*lmo1118*	AAAAGTTATTTGATGTTTCTGG	1393	1/2c and 3c	NC_003210	200
	TCAATTGCTTCAACGATAGA				200

**Table 3:** Serovar designations of strains studied by Brown *et al*. using different assays.

Strain	SRA accession	Sequence type	Clonal complex	Serovar according to:				
				Our assay	WGS	Agglutination	ELISA	DS
PNUSAL001711	SRS1068411	124	124	1/2a	1/2a	1/2a	ND	1/2a-1/2b
SKB427	SRS2772091	912	912	1/2a	1/2a	1/2a	ND	1/2a-1/2b
SKB429	SRS15800321	912	912	1/2a	1/2a	ND	ND	1/2a-1/2b
SKWL57	SRS8215092	912	912	1/2a	1/2a	ND	ND	1/2a/3a
SKWL62	SRS8224581	912	912	1/2a	1/2a	ND	ND	1/2a/3a
SKWL69	SRS8215188	912	912	1/2a	1/2a	ND	ND	1/2a/3a
SKWL223	SRS8205717	912	912	1/2a	1/2a	ND	ND	1/2a-1/2b
JJ25	SRS15801912	1055	1055	1/2a	1/2a	ND	ND	1/2b/3b
SKB57	SRS8205774	1055	1055	1/2a	1/2a	1/2a	ND	1/2b/3b
SKWL79	SRS8215344	1055	1055	1/2a	1/2a	1/2a	ND	1/2b/3b
PNUSAL000427	SRS531351	1082	1082	1/2a	1/2a	1/2a	ND	1/2b/3b
SKB102	SRS15800319	1365	1365	1/2a	1/2a	1/2a	ND	1/2a-1/2b
SKB128	SRS8205750	1365	1365	1/2a	1/2a	1/2a	ND	1/2a-1/2b
SKB397	SRS2772120	1383	912	1/2a	1/2a	1/2a	ND	1/2a-1/2b
SKWL256	SRS8215338	1492	1492	1/2a	1/2a	1/2a	ND	1/2b/3b
SKWL386	SRS15801917	1492	1492	1/2a	1/2a	ND	ND	1/2b/3b
SKWL388	SRS15801918	1492	1492	1/2a	1/2a	ND	ND	1/2b/3b
SKWL82	SRS8215330	1494	1494	1/2a	1/2a	1/2a	ND	1/2b/3b
SKWL85	SRS8215187	1494	1494	1/2a	1/2a	1/2a	ND	1/2b/3b
SKWL143	SRS8214724	1494	1494	1/2a	1/2a	1/2a	ND	1/2b/3b
SKWL155	SRS8215079	1494	1494	1/2a	1/2a	1/2a	ND	1/2b/3b
SKWL159	SRS15801913	1494	1494	1/2a	1/2a	ND	ND	1/2b/3b
SKWL347	SRS15801916	1494	1494	1/2a	1/2a	ND	ND	1/2b/3b
SKWL431	SRS15801919	1494	1494	1/2a	1/2a	ND	ND	1/2b/3b
SKWL39	SRS8205560	1503	1503	1/2a	1/2a	ND	ND	1/2a-1/2b
SKWL40	SRS15801914	1503	1503	1/2a	1/2a	ND	ND	1/2a-1/2b
CFSAN078591	SRS6197421	782	2	4b	4b	ND	4b	1/2b/3b
CFSAN081597	SRS6197237	782	2	4b	4b	ND	4b	1/2b/3b
CFSAN092770	SRS6199622	782	2	4b	4b	ND	4b	1/2b/3b
PNUSAL000395	SRS508797	782	2	4b	4b	4b	ND	1/2b/3b
PNUSAL001042	SRS716025	782	2	4b	4b	4b	ND	1/2b/3b
PNUSAL001307	SRS837087	782	2	4b	4b	4b	ND	1/2b/3b
PNUSAL001515	SRS978018	782	2	4b	4b	4b	ND	1/2b/3b
PNUSAL002350	SRS1596965	782	2	4b	4b	ND	ND	1/2b/3b
PNUSAL002394	SRS1618757	782	2	4b	4b	ND	ND	1/2b/3b
PNUSAL002732	SRS1893124	782	2	4b	4b	ND	ND	1/2b/3b
PNUSAL003280	SRS2647270	782	2	4b	4b	ND	ND	1/2b/3b
PNUSAL003431	SRS2681003	782	2	4b	4b	4b	ND	1/2b/3b
PNUSAL004174	SRS3644761	782	2	4b	4b	ND	ND	1/2b/3b
