# Supplementary material for: A Novel Sero-Specific-Gene Dependent Multiplex PCR Enhances the Discrimination of Major Listeria monocytogenes Serovars
Source: J Microbiol Biotechnol. 2025 Mar 7;35:e2411081. doi: 10.4014/jmb.2411.11081 (PMC11925748; doi:10.4014/jmb.2411.11081)
Supplement: Supplementary file 1 [file jmb-35-e2411081-supple.pdf]

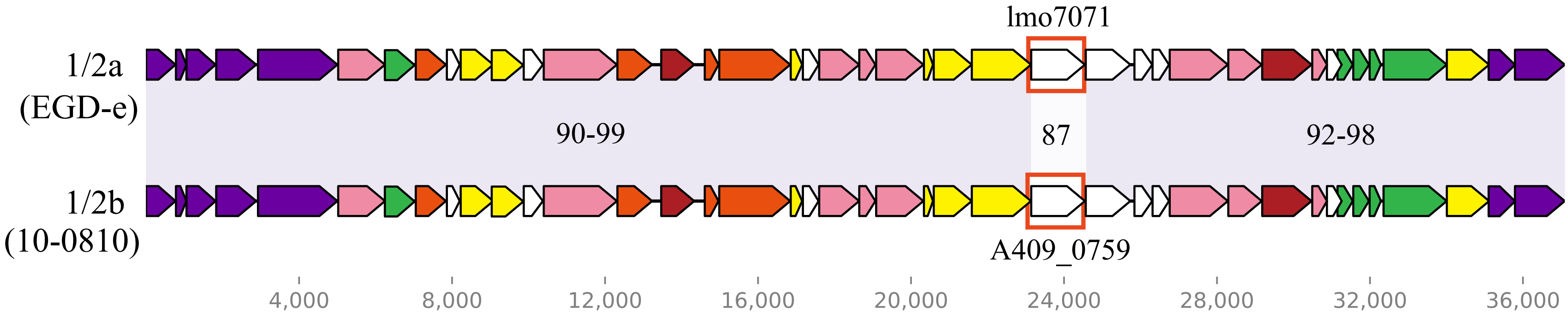

Fig. S1. Comparison of the flagellar gene clusters of *L. monocytogenes* serovar 1/2a and 1/2b.
Genes are catagorized by different color: pirple, genes encoding flagellar export apparatus; green, genes encoding basal body components; yellow, genes encoding motor/switch components; red, genes encoding proteins associated with chemotaxis; violet, genes encoding proteins involved in flagellar assembly; pink, genes encoding hook and junction proteins; white, genes encoding hypothetical proteins. % nucleotide identity were given, and sero-specific candidate genes were surrounded by red boxes.

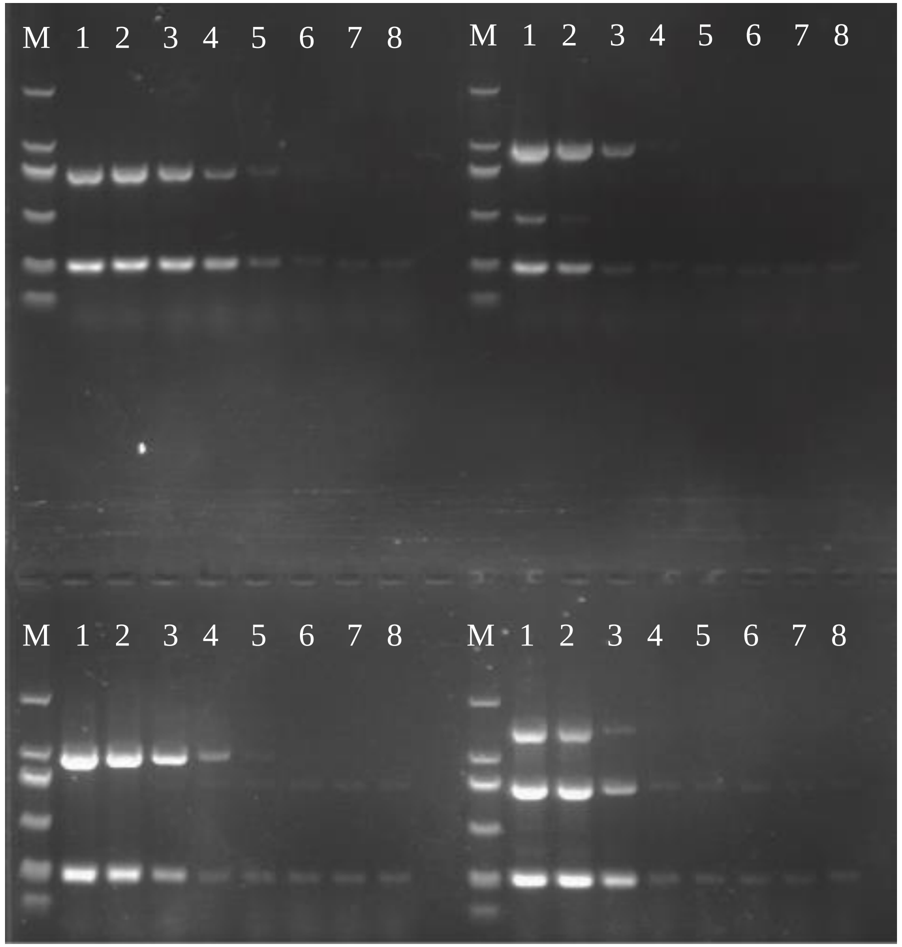

Fig. S2. Sensitivity evaluation of multiplex PCR revealed by 1.2 % agarose gel electrophoresis of the PCR products obtained from serially diluted genomic DNA of serovar 1/2a (top left), 1/2b (top right), 1/2c (bottom left), and 4b (bottom right). M, DL 2000 molecular standard; lane 1, 50ng; lane 2, 5ng; lane 3, 0.5ng; lane 4, 0.05ng; lane 5, 5pg; lane 6, 0.5pg; lane 7, 0.05pg; lane 8, 5fg.

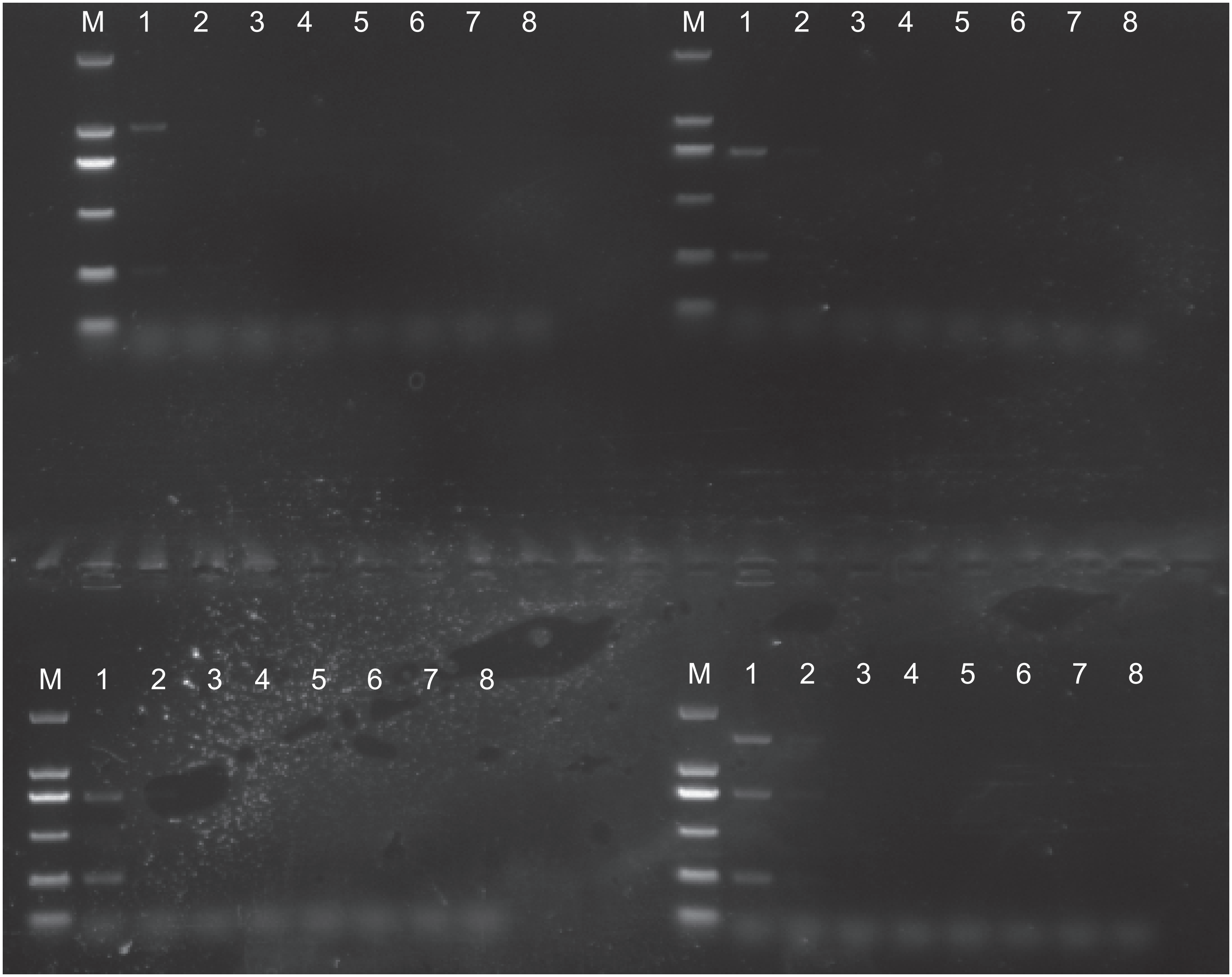

Fig. S3. Sensitivity evaluation of multiplex PCR revealed by 1.2 % agarose gel electrophoresis of the PCR products obtained from serially diluted bacterial culture of serovar 1/2a (top left), 1/2b (top right), 1/2c (bottom left), and 4b (bottom right). M, DL 2000 molecular standard; lane 1, 3×10^6 CFU; lane 2, 3×10^5 CFU; lane 3, 3×10^4 CFU; lane 4, 3×10^3 CFU; lane 5, 3×10^2 CFU; lane 6, 3×10^1 CFU; lane 7, 3 CFU; lane 8, 0 CFU.

Table S1. Serovar investigation of genomes from global isolates

Isolate	Accession	Serovar assigned by submitters	Serovar (our assay)	Serogroup (DS)	ST (MLST)	CC (MLST)	Note
CIIMS-NV-3	GCF_003409075.1	4b	4b	4b	328	CC1	
CLIP 2008/00154	ERR1100965	4b	4b	4b	10	CC1	
CLIP 2006/01023	ERR1100942	4b	4b	4b	1	CC1	
CLIP 2019/00525	ERR4998143	4b	4b	4b	1	CC1	
CLIP 2005/00172	ERR1100938	4b	4b	4b	1	CC1	
MRL-14-01208	ERR2519510	4b	4b	4b	1	CC1	
CLIP 2007/00596	ERR1100943	4b	4b	4b	1	CC1	
ERR2261420 TCsp31	ERR2261420	4b	4b	4b	1	CC1	
F2365	GCF_000008285.1	4b	4b	4b	1	CC1	
CLIP 2005/00008	ERR1100936	4b	4b	4b	1	CC1	
CLIP 2019/00553	ERR4998149	4b	4b	4b	1	CC1	
LL195	GCF_000318055.1	4b	4b	4b	1	CC1	
ERR2261425 TCsp31	ERR2261425	4b	4b	4b	1	CC1	
CLIP 2009/00277	ERR1100932	1/2a	1/2a	1/2a	101	CC101	
CLIP 2008/00396	ERR1100948	1/2a	1/2a	1/2a	621	CC11	
F6854	GCA_000167135.1	1/2a	1/2a	1/2a	11	CC11	
F6900	GCA_000168615.1	1/2a	1/2a	1/2a	11	CC11	
J2818	GCA_000168655.1	1/2a	1/2a	1/2a	11	CC11	
J0161	GCF_000168635.2	1/2a	1/2a	1/2a	11	CC11	
CLIP 2007/01014	ERR1100933	1/2a	1/2a	1/2a	121	CC121	
CLIP 2008/01050	ERR1100966	1/2a	1/2a	1/2a	121	CC121	
Lm-1704	SRR12560260	1/2a	1/2a	1/2a	121	CC121	
Lm-1264	SRR12560264	1/2a	1/2a	1/2a	121	CC121	
CLIP 2007/01398	ERR1100947	1/2a	1/2a	1/2a	121	CC121	
CLIP 2009/00373	ERR1100919	1/2a	1/2a	1/2a	121	CC121	
1425-35	ERR1432959	1/2a	1/2a	1/2a	121	CC121	
Lm-473	SRR12560266	1/2a	1/2a	1/2a	121	CC121	
Lm-568	SRR12560257	1/2a	1/2a	1/2a	14	CC14	
CLIP 2007/01377	ERR1100946	1/2a	1/2a	1/2a	14	CC14	
Lm-1263	SRR12560265	1/2a	1/2a	1/2a	155	CC155	
CLIP 2005/00065	ERR1100937	1/2a	1/2a	1/2a	155	CC155	

Lm16	GCF_002966935.1	1/2a	1/2a	1/2a	155	CC155
CLIP 2008/00235	ERR1100920	1/2a	1/2a	1/2a	155	CC155
Lm-1702	SRR12560261	1/2a	1/2a	1/2a	155	CC155
CLIP 2006/00727	ERR1100917	1/2a	1/2a	1/2a	18	CC18
CLIP 2007/01145	ERR1100945	1/2a	1/2a	1/2a	18	CC18
QOC2	GCF_000613165.1	1/2a	1/2a	1/2a	398	CC19
LM83088	ERR1100952	1/2a	1/2a	1/2a	19	CC19
LM07456	ERR1100964	1/2a	1/2a	1/2a	196	CC193
LM07423	ERR1100961	1/2a	1/2a	1/2a	193	CC193
LM07424	ERR1100962	1/2a	1/2a	1/2a	193	CC193
LM07425	ERR1100963	1/2a	1/2a	1/2a	193	CC193
CLIP 2006/01614	ERR1100929	1/2a	1/2a	1/2a	199	CC199
PNUSAL009635	GCA_017737415.1	4b	4b	4b	2928	CC2
CLIP 1989/13656	ERR1100934	4b	4b	4b	553	CC2
FSL J1-225	GCA_000212455.1	4b	4b	4b	290	CC2
HPB2262	GCA_000168815.1	4b	4b	4b	2	CC2
GMR-Lm-11-2020	ERR10441469	4b	4b	4b	2	CC2
CLIP 2009/00701	ERR1100970	4b	4b	4b	2	CC2
233	ERR3199915	4b	4b	4b	2	CC2
GMR-Lm-09-2020	ERR10441475	4b	4b	4b	2	CC2
CLIP 2009/00865	ERR1100909	4b	4b	4b	2	CC2
VIMHA007	GCF_001889645.1	4b	4b	4b	2	CC2
CLIST 3729	ERR3199917	4b	4b	4b	2	CC2
CLIST 3724	ERR3199920	4b	4b	4b	2	CC2
CLIP 2006/01244	ERR1100928	1/2a	1/2a	1/2a	20	CC20
CLIP 2009/00335	ERR1100931	1/2a	1/2a	1/2a	204	CC204
CLIP 2007/01067	ERR1100944	1/2b	1/2b	1/2b	386	CC224
CLIP 2007/00514	ERR1100927	1/2a	1/2a	1/2a	26	CC26
SLCC2755	GCF_000197755.2	1/2b	1/2b	1/2b	66	CC3
CLIP 2007/00948	ERR1100910	1/2b	1/2b	1/2b	3	CC3
CLIP 2007/00876	ERR1100935	1/2b	1/2b	1/2b	3	CC3
FSL R2-503	GCA_000168555.1	1/2b	1/2b	1/2b	3	CC3
LM07420	ERR1100958	1/2a	1/2a	1/2a	31	CC31
LM07422	ERR1100960	1/2a	1/2a	1/2a	31	CC31

LM07421	ERR1100959	1/2a	1/2a	1/2a	31	CC31
CLIST 3192	ERR3199919	4b	4b	4b	520	CC315
LM10-00505	ERR1100930	4b	4b	4b	194	CC315
CLIP 2009/01099	ERR1100926	1/2a	1/2a	1/2a	37	CC37
Lm-984	SRR12560251	1/2a	1/2a	1/2a	37	CC37
Lm-983	SRR12560252	1/2a	1/2a	1/2a	37	CC37
Lm-569	SRR12560256	1/2a	1/2a	1/2a	37	CC37
CLIP 2019/00520	ERR4998136	4b	4b	4b	388	CC388
CLIP 2019/00523	ERR4998138	4b	4b	4b	388	CC388
CLIP 2019/02984	ERR4998141	4b	4b	4b	388	CC388
CLIP 2019/00521	ERR4998137	4b	4b	4b	388	CC388
CLIP 2019/00527	ERR4998145	4b	4b	4b	388	CC388
CLIP 2019/00522	ERR4998139	4b	4b	4b	388	CC388
CLIP 2019/00517	ERR4998134	4b	4b	4b	388	CC388
CLIP 2019/00518	ERR4998135	4b	4b	4b	388	CC388
LM80459	GCF_000026705.1	4b	4b	4b	4	CC4
CLIP 2005/00190	ERR1100939	4b	4b	4b	4	CC4
CLIP 2008/01408	ERR1100967	4b	4b	4b	4	CC4
L31	GCA_006348975.1	4b	4b	4b	4	CC4
N2306	GCF_000950775.1	4b	4b	4b	4	CC4
CLIP 2009/00558	ERR1100911	4b	4b	4b	4	CC4
L312	GCF_000307085.1	4b	4b	4b	4	CC4
QOC1	GCF_000689335.1	1/2a	1/2a	1/2a	403	CC403
MRL-14-00748	ERR2519497	1/2a	1/2a	1/2a	403	CC403
CLIP 2007/01345	ERR1100925	1/2a	1/2a	1/2a	620	CC412
CLIP 2005/00704	ERR1100912	1/2b	1/2b	1/2b	5	CC5
CLIP 2007/01337	ERR1100924	4b	4b	4b	54	CC54
CLIP 2006/00918	ERR1100941	1/2b	1/2b	1/2b	59	CC59
CLIP 2007/01042	ERR1100918	1/2b	1/2b	1/2b	59	CC59
CLIP 2006/00444	ERR1100921	1/2b	1/2b	1/2b	59	CC59
CLIP 2006/00581	ERR1100954	4b	4b	4b	615	CC6
N13-0703	GCF_003265125.1	4b	4b	4b	6	CC6
CLIP 2009/01092	ERR1100951	4b	4b	4b	6	CC6
CLIP 2009/00372	ERR1100913	4b	4b	4b	6	CC6

142336	GCF_018380625.1	4b	4b	4b	6	CC6
CLIP 2007/01481	ERR1100914	1/2a	1/2a	1/2a	624	CC7
MRL-15-01290	ERR2519548	1/2a	1/2a	1/2a	624	CC7
SLCC98	ERR1100953	1/2a	1/2a	1/2a	98	CC7
LM73068	ERR1100974	1/2a	1/2a	1/2a	98	CC7
10403S	GCF_000168695.2	1/2a	1/2a	1/2a	85	CC7
SLCC5850	GCF_000307045.1	1/2a	1/2a	1/2a	12	CC7
CLIST 2140	ERR3199887	1/2a	1/2a	1/2a	12	CC7
EGD	GCF_000582845.1	1/2a	1/2a	1/2a	12	CC7
CLIP 2009/00261	ERR1100949	1/2a	1/2a	1/2a	7	CC7
CLIP 2008/01516	ERR1100923	1/2b	1/2b	1/2b	77	CC77
MRL-14-00018	ERR2519479	1/2a	1/2a	1/2a	1602	CC8
08-5578	GCF_000093125.2	1/2a	1/2a	1/2a	292	CC8
08-5923	GCF_000022925.1	1/2a	1/2a	1/2a	120	CC8
CLIP 2008/01457	ERR1100915	1/2a	1/2a	1/2a	8	CC8
CLIP 2005/00401	ERR1100940	1/2a	1/2a	1/2a	8	CC8
Lm-405	SRR12560267	1/2a	1/2a	1/2a	8	CC8
CLIP 2006/01598	ERR1100922	1/2b	1/2b	1/2b	87	CC87
FSL J1-194	GCA_000168395.1	1/2b	1/2b	1/2b	88	CC88
CLIP 2006/00983	ERR1100955	1/2a	1/2a	1/2a	391	CC89
CLIP 2009/00942	ERR1100971	1/2c	1/2c	1/2c	622	CC9
CLIP 2009/00432	ERR1100969	1/2c	1/2c	1/2c	441	CC9
CLIP 2009/00521	ERR1100916	1/2c	1/2c	1/2c	441	CC9
SLCC2372	GCF_000210815.2	1/2c	1/2c	1/2c	122	CC9
Lm-570	SRR12560255	1/2c	1/2c	1/2c	9	CC9
LM57179	ERR1100972	1/2c	1/2c	1/2c	9	CC9
Lm-970	SRR12560253	1/2c	1/2c	1/2c	9	CC9
CLIP 2009/01004	ERR1100950	1/2c	1/2c	1/2c	9	CC9
Lm-1705	SRR12560259	1/2c	1/2c	1/2c	9	CC9
LM77097	ERR1100975	1/2c	1/2c	1/2c	9	CC9
Lm-1262	SRR12560250	1/2c	1/2c	1/2c	9	CC9
LM6186	ERR1100973	1/2c	1/2c	1/2c	9	CC9
LM80661	ERR1100976	1/2c	1/2c	1/2c	9	CC9
Lm-914	SRR12560254	1/2c	1/2c	1/2c	9	CC9

Lm-1706	SRR12560258	1/2c	1/2c	1/2c	9	CC9	
Lm-1267	SRR12560262	1/2c	1/2c	1/2c	9	CC9	
Lm-1265	SRR12560263	1/2c	1/2c	1/2c	9	CC9	
FSL N3-165	GCA_000168535.1	1/2a	1/2a	1/2a	222	CC90	
LM07416	ERR1100957	1/2a	1/2a	1/2a	13	ST13	
LM07414	ERR1100956	1/2a	1/2a	1/2a	13	ST13	
2017-TE-6913-1	GCF_013122235.1	4b	4b	IVb-v1	218	CC218	
1282	ERR3199916	4b	4b	IVb-v1	218	CC218	
74	ERR3199918	4b	4b	IVb-v1	218	CC218	
19	ERR3199909	1/2c	4b	IVb-v1	218	CC218	
CLIP 2019/02983	ERR4998140	1/2a			1618	CC127	Listeria ivanovii
CLIP 2019/00524	ERR4998142	4b			1618	CC127	Listeria ivanovii
CLIP 2019/00528	ERR4998146	4b			1618	CC127	Listeria ivanovii
CLIP 2019/02979	ERR4998150	1/2a			1610	CC529	Listeria innocua
CLIP 2019/02982	ERR4998152	1/2a			603	CC660	Listeria innocua
CLIP 2019/02980	ERR4998151	4b			603	CC660	Listeria innocua
CLIST 3869	ERR3199907	1/2b	4b	4b	1	CC1	
CLIST 3865	ERR3199906	1/2b	4b	4b	1	CC1	
7	ERR3199904	1/2b	1/2c	1/2c	9	CC9	
CLIST 2137	ERR3199886	1/2a	1/2c	1/2c	9	CC9	
FSL N1-017	GCA_000168515.1	4b	1/2b	1/2b	3	CC3	
CLIP 2019/00526	ERR4998144	4b	1/2a	1/2a	155	CC155	
CLIP 2019/00531	ERR4998147	4b	1/2a	1/2a	155	CC155	
CLIP 2019/00529	ERR4998148	4b	1/2a	1/2a	155	CC155	
CLIP 2009/00402	ERR1100968	4b	1/2b	1/2b	5	CC5	
CLIST 3739	ERR3199899	4b	1/2b	1/2b	228	CC228	

Table S2. Species prediction of the genomes from global isolates those cannot be serotyped by our assay using Kmerfinder

#Template: ERR4998140	Num	Score	Expected	Template_l	Query_Cov	Template_(	Depth	tot_query_	tot_templa	tot_depth	q_value	p_value
NZ_LR134394.1 Listeria ivanovii subsp. londoniensis strain NCTC12701 genome assembly, chromosome: 1	17311	92868	1	106103	86.94	88.52	0.88	86.94	88.52	0.88	92862.6	1.00E-26
NZ_CP009576.1 Listeria ivanovii subsp. londoniensis strain WSLC 30151, complete genome	12141	3304	20	107470	3.09	3.09	0.03	85.34	85.57	0.85	3241.92	1.00E-26
#Template: ERR4998142	Num	Score	Expected	Template_l	Query_Cov	Template_(	Depth	tot_query_	tot_templa	tot_depth	q_value	p_value
NZ_LR134394.1 Listeria ivanovii subsp. londoniensis strain NCTC12701 genome assembly, chromosome: 1	17311	92914	1	106103	88.85	88.61	0.88	88.85	88.61	0.88	92909.41	1.00E-26
NZ_CP009576.1 Listeria ivanovii subsp. londoniensis strain WSLC 30151, complete genome	12141	3238	20	107470	3.1	3.03	0.03	87.04	85.48	0.85	3176.67	1.00E-26
#Template: ERR4998146	Num	Score	Expected	Template_l	Query_Cov	Template_(	Depth	tot_query_	tot_templa	tot_depth	q_value	p_value
NZ_LR134394.1 Listeria ivanovii subsp. londoniensis strain NCTC12701 genome assembly, chromosome: 1	17311	92901	1	106103	87.49	88.52	0.88	87.49	88.52	0.88	92895.96	1.00E-26
NZ_CP009576.1 Listeria ivanovii subsp. londoniensis strain WSLC 30151, complete genome	12141	3298	20	107470	3.11	3.09	0.03	85.89	85.58	0.85	3236.26	1.00E-26
#Template: ERR4998150	Num	Score	Expected	Template_l	Query_Cov	Template_(	Depth	tot_query_	tot_templa	tot_depth	q_value	p_value
NC_003212.1 Listeria innocua Clip11262 complete genome	13902	78187	4	103008	75.8	76.98	0.76	75.8	76.98	0.76	78174.44	1.00E-26
NZ_CP071157.1 Listeria innocua strain LI203 chromosome, complete genome	11462	7141	18	100371	6.92	7.23	0.07	75.02	78.53	0.77	7087.02	1.00E-26
NZ_CP071179.1 Listeria innocua strain LI47 chromosome, complete genome	14669	3166	19	100161	3.07	3.15	0.03	75.6	78.27	0.78	3109.32	1.00E-26
NZ_CP065605.1 Listeria welshimeri strain FDAARGOS_947 chromosome, complete genome	1434	2167	19	100795	2.1	2.16	0.02	52.99	54.51	0.54	2109.64	1.00E-26
NZ_CP050028.1 Listeria monocytogenes strain 4057 chromosome, complete genome	10745	1322	20	107532	1.28	1.22	0.01	19.73	18.83	0.19	1260.4	1.00E-26
NZ_CP045743.1 Listeria innocua strain CFSAN044836 chromosome, complete genome	22573	1145	19	97838	1.11	1.18	0.01	75.41	79.92	0.8	1089.23	1.00E-26
NZ_CP030102.1 Listeria monocytogenes strain FDAARGOS_57 chromosome, complete genome	16834	546	0	1243	0.53	43.69	0.44	0.66	55.22	0.55	545.29	1.00E-26
#Template: ERR4998151	Num	Score	Expected	Template_l	Query_Cov	Template_(	Depth	tot_query_	tot_templa	tot_depth	q_value	p_value
NZ_CP025202.1 Listeria innocua strain WSLC_2021_parental chromosome, complete genome	7545	94925	0	98809	94.83	97.12	0.96	94.83	97.12	0.96	94922.54	1.00E-26
NZ_CP050028.1 Listeria monocytogenes strain 4057 chromosome, complete genome	10745	1767	20	107532	1.77	1.64	0.02	20.31	18.83	0.19	1705.16	1.00E-26
NZ_CP030102.1 Listeria monocytogenes strain FDAARGOS_57 chromosome, complete genome	16834	235	0	1243	0.23	19.13	0.19	0.37	30.74	0.3	234.29	1.00E-26
#Template: ERR4998152	Num	Score	Expected	Template_l	Query_Cov	Template_(	Depth	tot_query_	tot_templa	tot_depth	q_value	p_value
NZ_CP025202.1 Listeria innocua strain WSLC_2021_parental chromosome, complete genome	7545	94952	0	98809	94.82	97.16	0.96	94.82	97.16	0.96	94949.54	1.00E-26
NZ_CP050028.1 Listeria monocytogenes strain 4057 chromosome, complete genome	10745	1772	20	107532	1.77	1.64	0.02	20.31	18.84	0.19	1710.14	1.00E-26
NZ_CP030102.1 Listeria monocytogenes strain FDAARGOS_57 chromosome, complete genome	16834	237	0	1243	0.24	19.3	0.19	0.38	30.91	0.3	236.29	1.00E-26
